# Local causation

**DOI:** 10.1007/s11229-021-03272-8

**Published:** 2021-07-22

**Authors:** T. D. P. Brunet

**Affiliations:** Department of History and Philosophy of Science, University of Cambridge, Free School Lane, Cambridge, CB2 3RH UK

**Keywords:** Causation, Regularities, Counterfactuals, Locality, Sheaf models

## Abstract

The counterfactual and regularity theories are universal accounts of causation. I argue that these should be generalized to produce *local* accounts of causation. A hallmark of universal accounts of causation is the assumption that apparent variation in causation between locations must be explained by differences in background causal conditions, by features of the causal-nexus or causing-complex. The local account of causation presented here rejects this assumption, allowing for genuine variation in causation to be explained by differences in location. I argue that local accounts of causation are plausible, and have pragmatic, empirical and theoretical advantages over universal accounts. I then report on the use of presheaves as models of local causation. The use of presheaves as models of local variation has precedents in algebraic geometry, category theory and physics; they are here used as models of local causal variation. The paper presents this idea as stemming from an approach using presheaves as models of local truth. Finally, I argue that a proper balance between universal and local causation can be assuaged by moving from presheaves to fully-fledged sheaf models.

## Introduction

An account of causation is universal, I will say, if it is committed in any way to the following *universal assumption* (UA),

- (UA) If **C** is a cause of **E**, then **C** is a cause of **E** always and everywhere (at every space-time location **V**).

How this assumption should be interpreted of course depends on which account of causation we employ, since this determines how we define both the general causal relata types **C** and **E** and what it means to be “a cause”. However, both Pearl’s (2009) modern regularity account and Lewis’ (1973) counterfactual account of causation are universal. Perhaps more extant accounts of causation are universal in this sense; perhaps all are.^1^ I here concentrate on these two.^2^

Lewis pointed out that Hume defined causality twice, once by regularity and again by counterfactual dependence.

[W]e may define a cause to be an object followed by another, and *where* all the objects, similar to the first, are followed by objects similar to the second. Or, in other words, *where*, if the first object had not been, the second never had existed. –Hume, *The Enquiry concerning Human Understanding*, my emphasis

Lewis’s (1973) concern is that regularity theories developed on the basis of Hume’s first definition remain problematic, so he turns his attention to the second and the provision of a counterfactual theory of causation. Others, like Pearl (2009), have remained stalwart regularity theorists. These are different projects, nonetheless something is shared by both of Hume’s original definitions: they define causes locally. In each case, ‘a cause’ is defined *where* an object has an invariant relationship with subsequent objects; neither says that objects always and everywhere have the same invariants.^3^ I begin by arguing that the extant counterfactual and regularity accounts are universal.

On the counterfactual account, relations of counterfactual dependence between causes and effects are defined relative to the actual world, and the actual world includes everything that is spatio-temporally related. Since **C** is a cause of **E** iff there is a chain of counterfactual dependencies starting with **C** and ending with **E** (see Sect. 3.2.1), the counterfactual account can only model causation relative to a world “always and everywhere”, relative to maximally spatiotemporally related collections. The most locally fine-grained analysis of causation that can be provided, on the counterfactual account, defines this-worldly counterfactual truth relative to other-worldly truth. Moreover, this universality in the counterfactual account is reflected in the models used,^4^ that refer to a specific world or point of evaluation *w* for which no finer structure is provided. Put another way, the counterfactual account lacks the expressive power to define anything but universal causation.

On the regularity account, **C** is a cause of **E** iff events of type **C** regularly precede events of type **E**. According to Baumgartner (2008), “universal regularities among event types or factors constitute the primary analysans”. Indeed, it is not unusual to include universality as a criterion for regularities being causal (Anjum and Mumford 2018). But the problem is not mere analytic preference for universally quantified event-type level analyses of causation; the commitment of modern regularity accounts of causation to UA is not mere analytic preference. The problem is that locations are not events, they do not figure in regularities among event types, so they cannot be causes. Indeed, “**C** taking place (in a particular location **V**)” is an event, and all instances or tokens of event types are presumably spatio-temporally located like this. But on a regularity interpretation UA cannot be about event tokens; UA is about universal regularities among event types. Plainly, a token event (in a particular location **V**) *never* regularly precedes another token event, since neither recurs at all. Once we move from non-recurrent tokens to their types, we are able to define causes on the regularity account, but token locations are lost in the process. The regularity analysis may be in a better position, but not by much. While the counterfactual account must define a cause relative to an entire world, the regularity account does not define causes anywhere in particular. The regularity accounts can express local regularities, but they turn out not to be causal.

What then should we say when UA seems to fail, when causation appears to be irregular or counterfactually unstable across locations? Modifying Hume’s words, what account should we employ when one object is followed by another, and all the objects similar to the first are followed by objects similar to the second in location **V**, but not followed by them in another location **U**? Likewise, what account is available when, if the first object had not been in **V**, the second never had existed in **V**, but if it had not been in **U** the second would have existed in **U** all the same? Hume says at least what we in fact do in such cases: we explain them away.

[W]hen any cause fails of producing its usual effect, philosophers ascribe not this any irregularity in nature; but suppose, that some secret cause, in the particular structure of the parts, have prevented the operation.—Hume, *Of Probability*

Today we refer not to a “secret cause” but to a causal-nexus (Anjum and Mumford 2018), causing-complex (Baumgartner 2008) or homogeneous population (Cartwright 1979; Dupré and Cartwright 1988) of background causal conditions **B**, themselves variably present at locations, in situations or on occasions, and their variation explaining the differences in putative causal relationships. That is, confronted with apparently local causation, advocates of universal accounts can fall back on a *universal explanation* (UE) for apparent variation in causation.

- (UE) Whenever **C** is a cause of **E** in **V** and it appears that **C** is not a cause of **E** in some distinct **U**, there always exists some **B** such that **C**+**B** is a cause of **E** in **V** and $\lnot$**B** at **U**.

UE is a meta-theoretic principle that is a hallmark of universal accounts of causation. Put another way, it says that when **C** is *a* cause of **E** somewhere, then it can only appear to not be *a* cause of **E** elsewhere—it cannot actually fail to be a cause of **E** elsewhere (UA)—and this is accounted for by postulating some variation in another interacting cause **B** of **E**. Apparent causal variation is explained by causal insufficiency. Moreover, if **B** is not yet apparent but merely postulated to explain apparent variation in causation, then it is a Humean “secret cause.” UE itself is logically complex: it is universal and conditional, metaphysical, epistemic, ontological, and moreover, “locative” or topological. It refers to causes, apparent failures of causation, the existence of potentially unobserved event types, and to locations. To my knowledge there is no account of causation or model thereof that is all these things at once and so no account where UE appears as axiom; there are plenty of accounts where it enters into theory at the point that irregularities need explaining.

We find approximations of UE in treatments of Humean solutions to irregularity, or imperfect regularities. For instance,

[That striking a match is a cause of its catching fire] is not refuted by a struck match that does not catch fire. Whenever a match is struck, but fails to light, it may now be argued that – notwithstanding the striking – not all factors of the corresponding complex sufficient condition for lighting matches have been instantiated on the respective occasion.—Baumgartner (2008), pg. 4

A similar principle is given in Cartwright’s (1979) idea of the correct connection between laws of association and causal laws. Cartwright offers this in response to counterexamples against the idea that causes increase the probability of their effects, saying,

In all cases, the cause fails to increase the probability of its effects for the same reason: in the situation described the cause is correlated with some other causal factor which dominates in its effect...A cause must increase the probability of its effects – but only in situations where such correlations are absent.—Cartwright (1979), pg. 423

The correct connection is then established only in situations where background correlations are absent, in other words, where situations are causally homogenous with respect to effects. In relation to UE, we might specify these situations as those where *U* and *V* are causally homogeneous with respect to all causally relevant factors $\mathbf{B}$. Similarly, Cartwright’s (2004) later view of laws as regularities that are only ever true *ceteris paribus* might be interpreted, via UE, as the claim that **B** occurs in some such conditions, and that apparent local violations of laws should be explained by violations of those conditions.

In many cases of scientific explanation, where irregularities might plausibly be steps on the way to scientific discovery, UE is not necessarily a poor principle to adopt. To hold to the claim that **C** causes **E**, despite the apparent irregularity at some **U**, may often lead to fruitful investigations into neglected and relevant causal conditions **B**. So UE can appear as an expression of a healthy scientific attitude toward mounting cases of falsifications of causal claims. However, adopting UE in all cases unreservedly would be an overly strong buffer against degeneration of sciences assuming universal accounts of causation—especially when the postulated causal-nexuses, complexes, or homogeneous populations require a growing number of secret causes.

For Hume, moreover, UE is a philosophical *supposition* about causation and not a natural constraint on causality. Other suppositions are available, so we do not need to give up on causality to accept apparent irregularities or counterfactual unstabilities as genuine. When a cause fails of producing its usual effect, Hume makes us aware that philosophers *could* have ascribed this to irregularities in nature. Nothing requires us to adopt UE or guarantees that it will lead to fruitful investigations in all cases of scientific explanation where causation appears to be irregular or counterfactually unstable. Confronted by continued apparent failures of UA, we could have rejected it instead of adopting UE. The problem is then whether we ever *should* reject UA, and what an account and model of causation that *explicitly* allowed for local variation in causation should look like.

In distinction from universal accounts of causation, this paper defines and defends the idea that our accounts of causation should be *local*. An account of causation is local, I will say, if it is explicitly committed to the following *local assumption* (LA).

- (LA) **C** is locally a cause of **E** iff **C** is a cause of **E** in some location **U** and (potentially at least) **C** is not a cause of **E** is some distinct location **V**.

This form of LA assumes for simplicity that causation is all-or-nothing. This is treated in Sect. 3.2.1 using local counterfactual models of causation. If we take causation to come in degrees, the degree of causal dependence of **E** on **C** is local iff there is some location **U** where **E** has (at least potentially) a degree of causal dependence on **C** different from what it has in another region **V**. See Sect. 3.2.2 for regularity based local causation that can come in degrees. Note also that we can provide a corresponding allowance for *local explanation* (LE),

- (LE) There are cases where **C** is a cause of **E** in **V** and **C** is not a cause of **E** in some distinct **U**, and for which there is no **B** such that **C+B** is a cause of **E** in **V** and $\lnot$**B** in **U**.

Many of our causal claims are local. Examples range from everyday causal variation, where UE is easily satisfied, to persistent scientific puzzles for which concrete conditions **B** satisfying UE are unavailable. The effect of heat on the boiling of water varies by altitude; the effect of barometric pressure on precipitation varies by latitude; infectiousness of disease varies in space and time (Delamater et al. 2019); causes of soil conditions vary topographically (Webster 2000); solar emission of neutrinos varies seasonally (Glashow and Krauss 1987); some physical “constants” seem to vary across the cosmos (Webb et al. 2011; Uzan 2011). Universal accounts of causation can accept these as interesting cases, but must assume the apparent variation in causation reduces to variation in causal conditions or to error. A local account of causation involves no such assumption, however. It allows that some apparent variation in causation is what we might term irreducibly *de locus*—the most fundamental explanation we can provide may be differences in location.

The following section justifies this idea by detailing some advantages of having a local account of causation (Sect. 2), while the latter sections show how to provide general models of local causation (Sect. 3) and what the best such models should look like (Sect. 4). The modelling approach offered in this paper provides a rigorous, flexible and general way to reap the advantages offered in Sect. 2. A reader that is not yet sceptical, or who is interested only in the technical details of the model, may safely skip to Sect. 3, where semantics for local causal claims is presented prior to supplementary support for the sheaf theoretic approach in Sect. 4. The presentation of a semantics for local causation is done step-wise, presenting a non-causal and simplified (0th-order) account of local-truth and reviewing the requisite mathematical tools (Sect. 3.1), as a stage to the following semantics of local causation (Sect. 3.2). The major ideas are presented in the language of category theory, since that is the appropriate and most succinct format for them. Specifically, I argue that sheaves of causal-models are the appropriate semantic structures for models of local causation (Sect. 4). This, I argue in the last section, is because the gluing axiom of sheaves provides an alternative to universality in accounts of causation.

## Local causation: a plausible alternative

I said above that the regularity and counterfactual account cannot handle locality as they stand; I did not say that they could not handle locality with suitable modification. This paper presents a suitable modification. This section argues that this approach to local causation costs us little and purchases much.

Local causation does not cost us much because we can model local causation without rejecting either the regularity or counterfactual account. This provided we build additional structure on the models we already use (Lewis 1973; Pearl 2009). To this end I show how to form categories of these familiar causal models (Sect. 3.2), then define a *local causal model* as a functor which assigns a model from such a category to each open subset of some topological space, which is conceived as a collection of organized spatio-temporal locations (Sect. 3.1). These models then allow us the freedom to define causes within familiar universal accounts, while building in enough topological structure to substitute, as necessary, universal causal claims for local ones.

Before entering into further details of models, I begin with three benefits of a local account of causation, one “pragmatic”, one “empirical” and another “theoretical”. These are, briefly, that we can usefully deploy local causation in a number of cases, that local accounts of causation are empirically adequate and determinate, and that local accounts of causation are a generalization of universal accounts that permit greater flexibility in our construction of theories of causation. These are described in turn below.

The pragmatic reason to allow causation to be local is just that there are many cases where causation does appear to be local, and that science can sometimes carry on usefully without universal explanations. Local causation allows us to avoid postulating the background conditions suggested by UE. We may succeed in finding a universal background explanation for apparently local cases, but this is sometimes a Procrustean task that we can helpfully avoid. Every empirical investigation must get on with some presuppositions, and supposing brute facts of local variation (“irregularities”) in causation is a perfectly viable way to do normal science. Once we allow causation to be local, we can afford to assume locality on a case-by-case basis, as needed. We know well enough that the boiling of water over heat varies by altitude due to background conditions of atmospheric pressure—UE is easily satisfied. However, we may never explain cosmological variations in constants by a difference of causal conditions (Webb et al. 2011; Uzan 2011). But that does not prevent us from doing cosmology. Indeed, difference of location may sometimes be the only useful difference to rely upon.

The empirical reason to provide for local causation is familiar: underdetermination of any account or model of causation that is restricted to locally limited evidence for its universally general causal claims. Hume and many others conceive of this broad problem as temporal; as Stanford (2009) notes, empirical underdetermination is often understood as a limitation on science “by the evidence we happen to have *at present*”.^5^ However, underdetermination is also spatial. Science is limited by the evidence we happen to have *here and now*. A proper understanding of the limits of universally general scientific claims should therefore recognize that underdetermination is spatio-temporal or, more generally, that it is topological. Our empirical evidence is topologically constrained in ways that regularity and counterfactual accounts are not. We may believe that spatially unrestricted claims are true, but can only survey the local crows for blackness, only the local swans for whiteness, and only correlate local barometers with weather patterns. Sometimes we can do local science by comparing local observations gathered separately. We may compare ornithological or meteorological data about different geographies, topographies, climates, etc. Nonetheless, we will continually confront the underdetermination of universal causal theories, since the vast majority of observations relevant to non-local causal claims are not collected together in the locations where we do science.

Put another way, we know that our best access to causation is provided by epistemically limited procedures, so there could be no set of observations **O** sufficient to decide on all potential cases of apparently local causation.^6^ When there is some apparent difference in causation between locations **V** and **U**, a universalist is committed to the existence of some background and causally relevant condition **B** differing between them. Nonetheless, we do not have empirical access to all of the conditions at any location, but only to small finite sets of observations $O_{V}$ and $O_{U}$ for each location. Ideally, there would be some observation *b* differing between $O_{V}$ and $O_{U}$ and indicative of some causally relevant **B**. However, potential local variations in causation are not limited to the small and finite, so we may perpetually find that $O_{V} = O_{U}$ or that there is no such $b \in O_{V} {\setminus } O_{U}$.^7^ In that case, though both universal and local accounts of causation may be empirically adequate, only the local account is empirically determined: what it says is true is determined by, and only determined by, the phenomena it saves. Local accounts allow us to sacrifice universal generalization to purchase empirical adequacy and determinacy.^8^

There are advantages of a local account of causation beyond saving the phenomena. The theoretical reason to allow for local causation is that it provides us with theories that can do more than the universal ones available. Indeed, local causation is a *generalization* of universal causation—the latter is a special case where causation does not vary according to location. One advantage of this added generality is that it widens the acceptable responses to instances of irregularities. As Anjum and Mumford (2018, § 5.4) note, if we assume the rule “same cause, same effect” is essential to our notion of causation, then a difference in effect from what is apparently the “same cause” has only two acceptable responses: either there is a difference in the “causal set-up”,^9^ or there was an element of chance involved. Say that both of these (UE) explanations are *de re*—they are about the things, events, of cause and effect.^10^ A local account of causation allows, in addition, explanations *de locus*. When we move to modelling local causation, this manifests as allowing that causal claims or rules are *locally true*.

If the slogan “same cause, same effect” seems an endorsement of determinism, then a local account allows determinism to be locally true—it provides for determinism *de locus*. If the slogan evokes the idea of laws, consider a patchwork of local laws, such as speed limits—laws *de locus*. If we analyse the slogan as asserting that counterfactuals are true of their antecedent and consequent event descriptions at entire worlds (Lewis 1974, p. 560), then a local account allows that counterfactuals have different truth values at different parts of a world—counterfactual dependence *de locus*. If it is taken as a claim that generalizations must be invariant to certain interventions to count as causal (Pearl 2009, p. 25, and see Woodward 2005, p. 15 and p. 239), then a local account allows that interventions that hold within a location may differ between locations—invariance *de locus*.^11^ Once we allow for variation *de locus* in our account of causation, what remains is to provide a way of explicitly accounting for location in our models of causation. These are not theoretical options for universal accounts.

In a model theoretic sense, we already treat causation this way. Causal statements are modelled or interpreted and are true only *relative* to models or interpretations. Half of the work is done; we have only to provide a satisfactory way of mapping locations to models, and we thereby obtain a localization of causal statements. When Bell (1986) or Goldblatt (1984) advocate a “local” interpretation of mathematics or “local truth”, it is in this model theoretic sense: different theorems of classical mathematics are validated in different model topoi—and this has surely been a theoretically fruitful sort of locality. In words modified, with homage, from Bell’s (1986) account of local mathematics: With the relinquishment of the absolute universe of causation, causal concepts will in general no longer possess absolute meaning, nor causal assertions absolute truth-values, but will instead possess meanings or truth-values only locally (p. 409). Indeed, far from hampering causal inference, the replacement of absolute by local causation results in a considerable gain in flexibility of application of causal ideas (p. 425).

## Local semantics

The term ‘sheaf’ stems from work by french mathematicians Jean Leray and Henri Cartan^12^ where the concept was put to work at a very different and far more sophisticated purpose than required of it here. It is a translation of the french word *faisceau*, itself from the latin *fascis*, variously translated as “bundle”, “beam”, “ray”, “cluster” and “sheaf”—all of which are distinct mathematical terms of art. The appropriate visual analogy is with fields of wheat, where stalks of wheatgrass are bundled together with spikes facing upwards and sit somewhere on the topography of the landscape—as depicted in Van Gogh’s *Sheaves of Wheat*. Further to the analogy, in a landscape of sheaves blowing in the wind, each section of the field may oscillate differently, encoding causal information about local gusts. This section supports this analogy with the formal resources of categorial sheaf-theory.^13^

This section begins by providing a generic account of local-truth (Sect. 3.1) then moves to exposition of local causation (Sect. 3.2), only in hopes that the latter seems a shorter leap from the former. There is a rich and diverse history of philosophical and logical work providing systems to handle locality. Too much to provide due credit. These systems are variously described as temporal logic (Prior 1957, 1968; Rescher 1968; Rescher and Urquhart 2012), spatial logic, topological logic (Garson 1973), prepositional logic (Garson 1981), locative logic, place logic and logic of location (Simons 2006). The sheaf theoretic approach used here arguably begins with Kripke’s (1965) models of intuitionism, where functors are used to organize possible states of knowledge according to a posetal ordering of time (see Goldblatt 1984, Ch. 8.4), the geometric modalities of, e.g., Goldblatt (1984, Ch. 14), and with the “local” accounts of truth in Bell (1986).

Within this diversity of presentations there is a corresponding diversity of syntaxes, metaphysical justifications and axiomatic systems. I will not here be concerned with which of these is correct—which metaphysics of tense or space we should adopt, which axioms about location or tense we should accept or which syntax to use—but instead focus on semantics only.^14^ The aim of this article is not to decide on an approach to location relative truth-values but to rely on such an approach to discuss location relative causation. To that end, only the most general features of a logic of location will be required.

I approach this in the language of category theory, since that is the appropriate setting for the analysis of (pre)sheaves. J. W. Gray (1979) attributes the following suggestive phrase to M. Auslander, “sheaf theory is the subject in which you do topology horizontally and algebra vertically”, with the afterthought that perhaps logic would be done in the third dimension. Presheaves are often represented as a horizontal plane depicting a topological space with algebraic structures such as rings or groups sitting above points or opens sets. My suggestion is that, while we continue to do topology horizontally, we use sheaf theory as a guide to doing causal modelling vertically. That is, to conceive of local causal models as presheaves, where causal models take the place of algebras.

### A local account of truth

In any local account of truth there are evidently three things that must somehow be associated semantically: (1) locations, (2) statements and (3) truth-values. Simons (2006) presents a general framework for logic of location that I will take as the point of departure here.^15^ Suppose that we have some collection **L** of *locations* (not otherwise specified) and a collection **S** of *statements*. Simons (2006) then defines a “truth-value distribution of **S** over **L**” to be a function $D{:}\, \mathbf{L } \times \mathbf{S } \rightarrow \{0,1\}$. The intuitiveness of this presentation is laudable: *D* assigns truth-values to pairs of a location and statement. I will argue for an essentially similar approach below. Though, to avoid confusion later on, let us first make the following substitutions. Instead of a collections of statements **S** consider an (arbitrary, 0th-order) language $\mathcal {L}$, and instead of a set of locations **L** let us consider a topological space $\tau$. Where $\varOmega = \{0,1\}$ is the set of truth-values, Simons’s (2006) proposal can then be re-written as a function $D{:}\, \tau \times \mathcal {L} \rightarrow \varOmega$, i.e., as a function from pairs of open-sets in $\tau$ and sentences of $\mathcal {L}$ to truth-values.

**Fig. 1:**
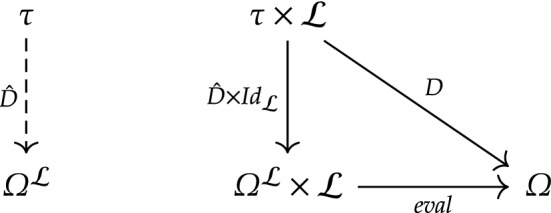
Commuting diagram for $\varOmega ^{\mathcal {L}}$ as a power object

By considering the domain of these semantic objects to be a product of location and sentence, this approach obscures something fundamental about local-truth: that it is a functor. Instead, I suggest we consider functions $\hat{D}{:}\,\tau \rightarrow \varOmega ^{\mathcal {L}}$, assigning to each open-set in $\tau$ some function $v{:}\, \mathcal {L} \rightarrow \varOmega$, i.e., assigning $\mathcal {L}$-models to $\tau$-locations. This is a change of perspective, not of fundamental concept. First, notice that $\varOmega ^{\mathcal {L}}$ is a power object, and is essentially a collection of truth-value functions (typically denoted $\mathbf{V}$). This implies that there is an isomorphism between $\hat{D}$ and *D*. That is, there is an assignment of a unique $\hat{D}$ to each *D*—where $\hat{D}$ is called the *transpose* or *currying* of *D*. According to the commuting diagram below: $D = \text {eval} \circ (\hat{D} \times Id_{\mathcal {L}})$ (Fig. 1).

The function *eval* simply applies the truth-value function in $\varOmega ^{\mathcal {L}}$ to a specific sentence in $\mathcal {L}$, giving a truth-value. This is logic as usual,^16^ except occurring at a specific open-set in $\tau$. I suggest using the transpose ${\hat{D}}$ of truth-value distributions *D* since, in this context, we are primarily concerned with the relationship between locations and models (truth-value functions), and not with the particular evaluations of sentences at locations. Moreover, this approach makes the definition of local-truth straightforward.^17^ Where $\mathbf{V}$ is a collection of $\mathcal {L}$-models, $\tau$ a topology, and $\mathcal {F}{:}\, \tau \rightarrow \mathbf{V}$ a function, we may define the truth of (an $\mathcal {L}$-sentence) $\alpha$ at a location $U \in \tau$.

$$\begin{aligned} \mathcal {F} \Vdash \alpha @ U \iff \mathcal {F}(U)(\alpha ) = 1 \end{aligned}$$

I will now advocate a categorial approach to local-truth before turning to local causation in the following section (Sect. 3.2). This first requires a category to serve as a collection of models. Consider the collection of 0th-order $\mathcal {L}$-models, i.e., the collection of truth-value functions $\mathbf{V}$ defined on $\mathcal {L}$. There is a collection of morphisms defined on $\mathbf{V}$. These we might call truth-value re-assignments and are functions $f:v_{i} \rightarrow v_{j}$ transforming one truth-value assignment $v_{i}$ into another $v_{j}$. These functions must at most re-assign truth values to atomics while otherwise *preserving recursion* in the definitions of truth for compounds. Together, these collections form a category $\mathfrak {V}$. We also need a categorial equivalent of a topology. Given a topological space $\langle \mathbf{X} , \tau \rangle$, there is a standard categorialization, $\mathcal {T}_\mathbf{X }$ having as objects the collection of open-sets $\tau$ and an arrow $f{:}\,V \rightarrow U$ whenever $V \subseteq U$.

A local ($\mathcal {L}$-)model can now be succinctly defined. $\mathcal {F}$ is a local ($\mathcal {L}$-)model iff $\mathcal {F}$ is a $\mathfrak {V}$-valued presheaf over $\mathcal {T}_\mathbf{X }$, i.e., $\mathcal {F}$ is a functor,

$$\begin{aligned} \mathcal {F}{:}\, \mathcal {T}\,\,_\mathbf{X }^{op} \rightarrow \mathfrak {V} \end{aligned}$$

Unpacking this definition into some of the terminology of sheaf theory, $\mathcal {F}$ is a functor such that,

1. For each open-set object *U* of $\mathcal {T}_\mathbf{X }$ there corresponds an (ordinary model) object $\mathcal {F}(U)$ of $\mathfrak {V}$, where $\mathcal {F}(U)$ is called *the sections of* $\mathcal {F}$ *over* *U*. Typically, $\mathcal {F}(U)$ is a set of some kind, hence the plural ‘*sections*’, and for our purposes it will suffice to consider it as a set of ordered pairs $\langle \ell , \omega \rangle$ such that $\ell \in \mathcal {L}$ and $\omega \in \varOmega$.
2. For each inclusion $f{:}\, V \rightarrow U$ there corresponds a truth-value re-assignment $\mathcal {F}(f):\mathcal {F}(U) \rightarrow \mathcal {F}(V)$. These are typically called *restriction morphisms*, i.e., the restriction of *U* to *V* and will here be denoted $\rho _{V,U}{:}\, \mathcal {F}(U) \rightarrow \mathcal {F}(V)$. In essence, whenever $V \subseteq U$, we are able to restrict or further localize our model $\mathcal {F}(U)$ to a model $\mathcal {F}(V)$ for the contained location *V*. These satisfy two constraints in addition: (1) $\rho _{U,U} = Id_{\mathcal {F}(U)}$ and (2) $\rho _{W,V} \circ \rho _{V,U} = \rho _{W,U}$ whenever $W \subseteq V \subseteq U$.

We should not now be concerned whether this is the correct or best model of local truth, but just that it is sufficiently general. In particular, it is a generalization of non-local, absolute truth. A “local” model $\mathcal {F}$ can nonetheless model a case in which truth is non-local when it is a constant pre-sheaf. That is, we obtain a non-local model from a local one by setting $\mathcal {F}(U_{i}) = v$ and $\rho _{U_{i}, U_{j}} = Id_{v}$ for all $U_{i}, U_{j} \in \tau$, so that $\mathcal {F}$ is essentially just the truth-value function *v*. Similarly, we can model a case where the truth is “different everywhere” by a pre-sheaf where $\mathcal {F}(U_{i}) = \mathcal {F}(U_{j}) \implies U_{i} = U_{j}$.

Before moving to an account of local causation, we should cover the conditions that a pre-sheaf must satisfy to be a sheaf. Firstly, because this will go easier without the additional complexities of modelling causation, and secondly because it is sheaves, I will argue, that *best* capture local causation (Sect. 4). Consider an example motivating sheaves in the context of local-truth.

There are 25 bridges in the city of Cambridge, 10 in the Middle River district and 1 on the grounds of Clare College. To be a pre-sheaf, a local model of claims about the number of bridges by location must specify these numbers, but it must also tell us how, when in Cambridge, we are to properly restrict our claims to both the Middle River and to Clare College. Moreover, it should give us the same answer whether we restrict ourselves to Clare College directly, or first via Middle River. This is intuitive enough, indeed it borders on what we would trivially expect from considering smaller and smaller sections of a map. But consider that it *does not* specify what should obtain when we compare maps along overlapping edges. It is possible to cover Cambridge by a collection of maps, none of which themselves have 25 bridges. Indeed, pre-sheaves do not in general guarantee that we can come to any conclusions by integrating or gluing together compatible information about given locations. Sheaves, however, do.

To define a sheaf we also require the notion of an *open cover*, which is an (indexed) family of sets $\{U_{i}\}$ such that $U \subseteq \bigcup _{i \in I} U_{i}$. That is, a set is a subset of the union of an open cover of it. A *sheaf* is then a presheaf $\mathcal {F}$ satisfying two additional constraints relating sections to open coverings in $\langle \mathbf{X} , \tau \rangle$. These conditions are called (1) *locality* and (2) *gluing*.

1. **Locality** If $s,t \in \mathcal {F}(U)$ are such that $\rho _{U_{i},U}(s) = \rho _{U_{i},U}(t)$ for all $U_{i}$ covering *U*, then $s = t$. In other words, sections are uniquely determined “locally”, by their restrictions.
2. **Gluing** If $\rho _{U_{i} \cap U_{j}, U_{i}}(s_{i}) = \rho _{U_{i} \cap U_{j}, U_{j}}(s_{j})$ for all *i*, *j*, then there exists a (unique) $s \in \mathcal {F}(U)$ such that $\rho _{U_{i}, U}(s) = s_{i}$ for all *i*. When restriction of sections “agree” or are “compatible” on intersections of underlying locations we are assured the existence of a more global section obtained by concatenating or *gluing* these together.

Presentations of the definition of sheaves are quite diverse.^18^ We can thankfully here concentrate on them in this simplified and limited context. Recall that a section $s \in \mathcal {F}(U)$ is a pair $s = \langle \ell _{s}, \omega _{s} \rangle$ of a sentence $\ell _{s} \in \mathcal {L}$ and a truth-value $\omega _{s} \in \varOmega$. *Locality* then says just that, for *distinct* pairs $s \ne t$ there is some sublocation where they are re-evaluated differently. That is, moving from a pre-sheaf to a sheaf comes with the additional constraint that distinct truth-value assignments must be distinctly re-evaluated at some sublocation.

*Locality* tells us something about truth-evaluations moving more locally; *gluing* tells us about what happens as we move to a more global evaluation. Consider the simplest case, where we have some *U* and *V*, and imagine that $U \cap V \ne \emptyset$. *Gluing* says that, if we find compatible evaluations, i.e., a sentence that is re-assigned the same truth-value in $U \cap V$ whether this is done from *U* or from *V*, then there is an assignment of that sentence in $U \cup V$ which is, from there, re-evaluated to what it is in *U*. For an example, consider the weather being evaluated in the United Kingdom and in Ireland (which intersect in Northern Ireland and are together the British Isles). Being a pre-sheaf, we imagine that our local-model at least assigns some model of the weather to each location and some way of restricting that model to sublocations. Consider a particular statement about the weather, such as $\ell =$ “It rains most of the year.”. If our local model is a sheaf, we are guaranteed the following: if our re-evaluation of $\ell$ to Northern Ireland is the same from Ireland as it is from the United Kingdom, then there is some way of restricting our evaluation of $\ell$ in the British Isles to precisely what it is in Ireland. Compatible re-evaluations guarantee more-global evaluations. In Sect. 4 I argue that gluing, more than universalization, appropriately captures our best scientific account of causation.

### A local account of causation

But when different effects have been found to follow from causes, which are to *appearance* exactly similar, all these various effects must occur to the mind in transferring the past to the future, and enter into our consideration, when we determine the probability of the event... we must not overlook the other effects, but must assign to each of them a particular weight and authority. —Hume, *Of Probability*, 56

In his writing on probability, we find a somewhat different Hume. Instead of invariant causal relationships “which are entirely uniform and constant in producing a particular effect” [ibid], Hume considers those “more irregular and uncertain” cases—the purgative effect of rhubarb, the soporific effect of opium—and the consequent problem of accounting for observed irregularities in causation (see Gower (1991) and references therein). We are also given an example of *local variation of probabilities*,

It is more probable, in almost every country of Europe, that there will be frost sometime in January, than that the weather will continue open through out that whole month; though this probability varies according to climate, and approaches to a certainty in the more northern kingdoms.—Hume, *Of Probability*, 56

This variation is, however, not Hume’s primary target, since he continues: “[I]t seems evident, that, when transferring the past to the future... we transfer all the different events, in the same proportion as they have appeared *in the past*” [ibid, my emphasis]. Were Hume addressing climatologists, we can imagine that he would have placed more importance on what is different about this case, over and above irregularity and uncertainty. No amount of transferring the proportions of past events will suffice to correctly assess that probability—of observing frost given that it is January—if those past events were observed in different climates. An untravelled Englishman is ill equipped to be a Norwegian meteorologist.^19^

Rectifying this is straightforward: we must either restrict our assessment of the transfer of past proportions of events to a given location, or refine Hume’s claim about transferring the past to the future to say “...in the past and at a given location”—we must do “science locally” or do “local science”, respectively. These are similar but not the same. They entail different things about what is demanded of scientists and modellers of causation. If we only ever did science locally, then the problems that emerge from attempting to transfer our estimations about causation to other locations could be set aside and we could do science as usual. Doing “local science” instead entails that we account for location in the procedures of science. This immediately raises a question for modellers: *how should we account for location when modelling causation?*^20^

**Fig. 2:**
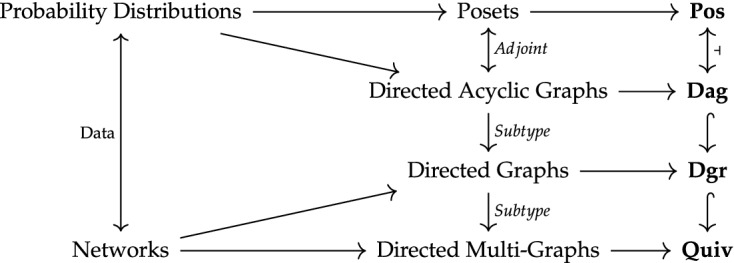
Informal diagram of the relationships between a selection of structures used to represent and model causation. On the far left, scientists represent data as either probability distributions or networks. The formal mathematical structures arising from these modelling tools occupy the middle block. Posets and DAGs are the formal tools most often associated with models of probability distributions. Posets are the objects of the category **Pos**, which is left adjoint to the category **Dag** of directed acyclic graphs. Corresponding to the fact that DAGs are a subtype of directed graphs, which are in turn a subtype of directed multi-graphs, the category **Dag** is a subcategory of the category **Dgr** of directed graphs, itself a subcategory of the category **Quiv** of quivers (which is a topos)

In this section I present the idea that introducing a functor based localization approach into causal modelling—analogous to that involved in modelling local-truth—is one way to go about accounting for location when modelling causation. Unlike the simplified case of propositional models $\varOmega ^{\mathcal {L}}$, there is a plurality of distinct types of models of causation used in science. While there is justifiable disagreement about this picture depending on variant definitions, the following informal diagram (Fig. 2) roughly describes the relationship between some common models of causation. Though not easily placed on the diagram, a further example of causal modelling using category theory deploys symmetric monoidal categories in models of quantum phenomena (Fong 2013; Coecke and Lal 2013).

Thankfully, there are many pursuits in science^21^ that have taken steps in the direction of localization of causes. Localization is used in spatial epidemiology (Elliott et al. 2000) and phylogeography (Soltis et al. 2006), where causal relationships are localized on the basis of disease contributing factors and patterns of evolution, respectively. Within physics, a program of research which uses partially ordered causal sets (Bombelli et al. 1987; Bombelli 1983; Sorkin 1991, 2005) has been integrated with a localization approach based on functors (Raptis 2000, 2001; Mallios and Raptis 2001). The use of (pre)sheaves of probability distributions (Abramsky and Brandenburger 2011) has been advanced as models of ‘non-locality’ (Bell 1964) and contextuality (Kochen and Specker 1975) of quantum measurements. Though without sheaf-theoretic framing, Cavalcanti’s (2018) approach to non-locality and contextuality using Pearl’s causal models, is yet another case where localization of causes has been fruitfully applied in physical science. In some of these physical cases, the mathematical models used are fully-fledged *sheaves*. Though each are embroiled in the analysis of the particulars of their target systems, these programmes can be given a local character when categories of the corresponding causal models are used as values for presheaves.

We do not lack material for local causal modelling; we have a disunified abundance. Given this diversity, we should begin with a very general idea about how causation can be localized, then impose constraints or specifications on this method depending on our particular view of causation and locations. As Pearl says of general theories of causation,

In addition to embracing all questions judged to have causal character, a general theory must also subsume any other theory or method that scientists have found useful in exploring the various aspects of causation. In other words, any alternative theory needs to evolve as a special case of the “general theory” when restrictions are imposed on either the model, the type of assumptions admitted, or the language in which those assumptions are cast. —Pearl (2010)

For questions with a *local* causal character, Sect. 3.1 provides such a general theory. A *local causal model* is going to be, in general, an $\mathcal {S}$-valued presheaf over some topological category $\mathcal {T}_{X}$, where $\mathcal {S}$ is a category the objects of which are “causal models” and the morphisms of which are appropriate model-structure preserving maps. What remains is to specify the category $\mathcal {S}$, and the appropriate morphisms, for causal claims cast in the languages of counterfactuals and regularities.

#### Local counterfactuals

I use the counterfactual account of causation of Lewis (1973) for simplicity and because the advantages and shortcomings of this account are well known (Salmon 1994; Woodward 2005; Menzies and Beebee 2001; Collins et al. 2004). There seems to be an emerging consensus that the counterfactual account of causation is not the most useful for scientists. Nonetheless, as Lewis (1973) points out, “we do know that causation has something or other to do with counterfactuals.” To define counterfactual dependence  between two sentences *A* and *B* we require something like a Kripke-model $\mathfrak {M} = \langle \mathfrak {F}, w \rangle$, where $\mathfrak {F} = \langle W, R \rangle$ is like a Kripke-frame and *W* is a set of “worlds” (or “points”) as usual, except that the accessibility relation $R \subseteq W \times W \times W$ is *trinary* and interpreted as a nearness or similarity relation. Lastly we assign an entailment relation to each world $w \mapsto \ \Vdash _{w}$. Lets call these Lewis-models. Next we define the entailment of counterfactual dependence by the model as,That is, *B* is counterfactually dependent on *A* iff for every world that makes A true and B false, there is a closer (more similar but not identical) world that makes both true—or, it takes more deviation from the world *w* to make A not imply B than it does to make A imply B. The next step is to “extend causal dependence to a transitive relation in the usual way” (Lewis 1973, p. 563). Given a language $\mathcal {L}$ for which $\mathfrak {M}$ is a model, where $C_{i}...C_{n} \in \mathcal {L}$, define,Intuitively, we can read $A \leadsto B$ as “A is a cause of B”. Granted a language $\mathcal {L}$ extended by the rule,

If $\alpha , \beta$ are sentences, then $\alpha \leadsto \beta$ is a sentence.

The Lewis-model can then be treated as a semantic function $\mathfrak {M}{:}\, \mathcal {L} \rightarrow \varOmega$, as above (Sect. 3.1).

Notice that  follows trivially from the consistency of $\mathfrak {M}$: since there can be no world $w'$ where $\mathfrak {M} \Vdash _{w'} A \wedge \lnot A$. Secondly, counterfactual-dependence implies causation: , taking $n=1$ and $C_{1} = B$. Evidently, if $\mathfrak {M}$ is an $\mathcal {L}$-model, the poset  imposed by $\mathfrak {M}$ gives rise to a category with elements of $\mathcal {L}$ as objects and an arrow $f{:}\, \alpha \rightarrow \beta$ iff . But for the end of localizing causation it is not enough to recognize that Lewis models give rise to a posetal category. This categorial model of causation is still “universal” or “global”; it makes no mention of, nor dependency on location. Moreover, presheaves into this category would assign members of $\mathcal {L}$ to open sets, localizing only single causal claims, not causation.

To define a local counterfactual model we require a collection of Lewis-models and maps between these. We have fairly strong reasons regarding what exactly these maps should be. Firstly, the singleton world appearing in the model is the “actual” world, and the actual world should not vary from place to place. We should not be able to leave actuality by going some*where*. Secondly, a Kripke-frame is the model theoretic equivalent of what, according to Lewis (1986), we might call one particular way that the totality of all worlds is. If we assign exactly the *same* frame and actual world to each place, then there is no room for causation to vary either. Nonetheless, if we assign entirely different frames to different places within the same model, then we have metaphysically committed ourselves to places where the totality of all things *there* is different from what it is *here*. We cannot move the heavens, the best we can do is affect a shift in perspective on the constellations of possible worlds; we can rescue some metaphysical intuitiveness by stipulating that between frames there should at least be an invariance of the primitive relations of similarity among worlds. That is, that our maps between Lewis-models should be *actuality preserving and frame relation preserving maps*. Causation can then still vary between a model and those it maps to, simply because the assignment of an entailment to worlds still differs, while nonetheless actuality and the overall background structure of the “sum totality of everything” is preserved.

Happily, these sorts of maps are also perfectly well defined mathematically, once we consider what sort of set Lewis-models are. A Lewis-model is essentially a (trinary) *pointed-related set*, the morphisms of which form a category (Rydeheard and Burstall 1988; Adámek et al. 2004). A trinary pointed related set is an object $\langle A, R_{A} \subseteq A \times A \times A, *_{a} \in A \rangle$ where *A* is a set, *R* a 3-relation on *A* and $*_{a}$ some element of *A* selected as the point. If $\langle B, R_{B}, *_{b} \rangle$ is another such set, and $f{:}\, A \rightarrow B$ is a set-function, then *f* is a morphism of pointed related sets iff,

1. $$f(*_{a}) = *_{b}$$
2. $$R_{A}(x,y,z) \implies R_{B}(f(x), f(y), f(z))$$

Likewise, a morphism $f{:}\, \mathfrak {M}_{1} \rightarrow \mathfrak {M}_{2}$ of Lewis-models $\mathfrak {M}_{1} = \langle \mathfrak {F}_{1}, w_{1} \rangle$ where $\mathfrak {F}_{1} = \langle W_{1}, R_{1} \rangle$ (and $\mathfrak {M}_{2}$ likewise), is a function that is (1) *actuality preserving*: $f(w_{1}) = w_{2}$ and (2) *Kripke-relation preserving*: $R_{1}(w,w',w'') \implies R_{2}(f(w), f(w'), f(w''))$. Moreover, this sort of mapping allows that  while , since there is no stipulation that maps preserve assigned entailment relations, that $\Vdash _{w^{i}} \ = \ \Vdash _{f(w^{i})}$, for arbitrary $w^{i}$. Call these ‘Lewis re-modellings’, by analogy with the sense of re-assignments involved in local-truth. For our purposes, this defines the category $\mathfrak {Lew}$ having objects the Lewis-models and morphisms the actuality and relation preserving maps.

A local causal model can now be defined. $\mathcal {F}$ is a local causal model iff $\mathcal {F}$ is a $\mathfrak {Lew}$-valued presheaf over $\mathcal {T}_\mathbf{X }$.

$$\begin{aligned} \mathcal {F}\,{:}\, \mathcal {T}_\mathbf{X }^{\,\,\,op} \rightarrow \mathfrak {Lew} \end{aligned}$$

To each open set *U*, $\mathcal {F}$ assigns some Lewis-model $\mathcal {F}(U) = \mathfrak {M}_{U}$. This straightforwardly gives rise to a definition of local-truth of causal sentences, i.e.,

1 $$\begin{aligned} \mathcal {F} \Vdash (\alpha \leadsto \beta )@U \iff \mathcal {F}(U) \Vdash _{*_{\mathcal {F}(U)}} \alpha \leadsto \beta \end{aligned}$$

Before turning to regularity analysis and justifying a sheaf theoretic model for causation, I will simply spell out some consequences of this approach.

Treating the pre-sheaf $\mathcal {F}$ as a local causal model involves looking at Lewis-models $\mathfrak {M}_{U}$ as a *collection of sections* $\mathcal {F}(U)$ over *U*. The question naturally arises what then should be considered an individual *section* of $\mathcal {F}(U)$. Categorially, it is sufficient to say that an element is a function $x{:}\, \mathbf{1} \rightarrow \mathcal {F}(U)$ in $\mathfrak {Lew}$ from the terminal object $\mathbf{1}$. But the abstraction of this definition requires some unpacking. Firstly, the terminal object in $\mathfrak {Lew}$ is going to be any singleton of the form $\mathbf{1} = \langle \{*_{w}, w' \}, \{\langle *_{w},*_{w},w' \rangle \}, *_{w} \rangle$ since there is exactly one arrow $!{:}\, \langle A, R_{A}, *_{a} \rangle \rightarrow \mathbf{1}$ in $\mathfrak {Lew}$ from any other Lewis-model $\langle A, R_{A}, *_{a} \rangle$. This is defined by (1) $!(*_{a}) = *_{w}$ and (2) $\forall _{a \ne *_{a}} \ !(a) = w'$. Notice that **1** cannot be an object of the form $\langle \{0 \}, \{\langle 0,0,0 \rangle \}, 0 \rangle$, as it would be in the plain category of trinary pointed relations, since $\langle 0,0,0 \rangle$ is not a meaningful comparative similarity relation.

In other words, a section $s \in \mathcal {F}(U)$, an “element” of a Lewis-model, is another Lewis-model $s = \langle \{*_{w}, w' \}, \{\langle *_{w},*_{w},w' \rangle \}, *_{w} \rangle$ with a pair of worlds from the frame of the first, one actual and some other world, which are related in the only meaningful way (such that the actual world is more closely related to itself than it is to the other world). Assessing the truth of a non-trivial counterfactual sentence  relative to an element $s \in \mathcal {F}(U)$ is then just a matter of checking whether $\alpha \wedge \beta$ is true at the “other world”, i.e. whether $s \Vdash _{w'} \alpha \wedge \beta$. Put another way, while a Lewis-model typically considers a collection of alternative possibilities, the *elements* of a Lewis-model consider only a single alternative to actuality.

**Fig. 3:**
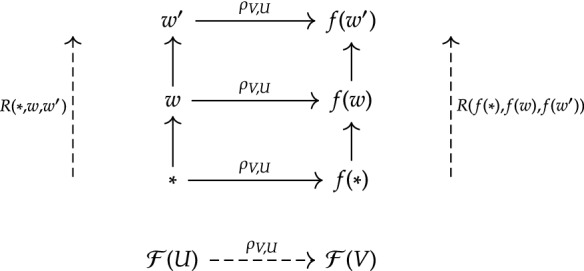
Depiction of restriction morphisms between Lewis-models. Note actuality- and relation-preservation

We can also give some sense to the restriction morphisms of *F*. Being a pre-sheaf, $\mathcal {F}$ not only provides a causal model for each location, it also provides a way of restricting our causal model—a way of re-modelling causation—at sub-locations. Graphically, we can picture how this must work for some worlds $*, w, w'$ of $\mathcal {F}(U)$ (Fig. 3). Here if $w \mapsto \Vdash _{w}$ differs from $f(w) \mapsto \Vdash _{f(w)}$, it can happen that $\mathcal {F}(U)$ and $\rho _{V,U}[\mathcal {F}(U)]$ differ in what is counterfactually true. For example, we might restrict ourselves to a location where, *from there*, the closest $\alpha$-world is no longer also a $\beta$-world.

To conclude the presentation of the case for local counterfactuals I will now show how to define such a presheaf by topologizing a “global” Lewis-model. Granted some model $\mathfrak {M} = \langle W, R, w \rangle$, since *W* is a set of some kind, we can consider a topology $\tau$ on *W* and a corresponding topological space $W_{\tau } = \langle W, \tau \rangle$. The purpose of doing this is firstly to provide some concrete $\mathcal {T}_{W}$, but also to generate a family of Lewis-models, one for each $U_{i} \in \tau$. To the latter end we require a “marking” function $*{:}\, \tau \rightarrow W$ assigning some actual world to each $U \in \tau$, satisfying,

1. $$*(U) \in U$$ for $$U \ne \emptyset $$
2. $$*(W) = w$$
3. $*(\emptyset ) = w$ (further trivial cases involving empty or singleton sets are hereafter ignored)

We can then form the family of Lewis-models,

2 $$\begin{aligned} \mathfrak {M}_{i} \ | \ \mathfrak {M}_{i} = \langle U_{i} \in \tau , R|_{U_{i}}, *(U_{i}) \rangle \end{aligned}$$

and the collection of morphisms of Lewis-models as above. Together these form a category $\mathfrak {Lew}_{\mathfrak {M}}$ of Lewis-submodels of $\mathfrak {M}$, providing for an evident pre-sheaf $\mathcal {F}{:}\, \mathcal {T}_{W}^{\,\,op} \rightarrow \mathfrak {Lew}_{\mathfrak {M}}$ where $\mathcal {F}(U) = \mathfrak {M}_{U}$ and restriction maps are morphisms of corresponding Lewis-models. Finally, $\mathcal {F}$ is a sheaf just when it is a sheaf of pointed related sets, provided the relation is meaningfully one of similarity.

#### Local regularities

Other accounts of causation require some other interpretations of restrictions and decisions about a suitable notion of invariance. Returning to Hume’s example of geographic variation, we may instead want restrictions to manifest as maps between conditional probability distributions, and for invariance to take the form of constraints on reconditionalizing averaged over the sum of contained locations. E.g., the probabilities that it will frost in January given one is in some region of England should average, over all subregions, to be the probability that it will frost in England overall. Today, however, we can do a bit better than Hume’s native regularity analysis.

Despite the problems facing both, Hume’s regularity analysis arguably survives in its most complete form today in the causal graph analysis of, e.g., Pearl (2009). Free somewhat from the metaphysical convolutions of Lewis-models, the definition and exposition of local causal models stems quite well from Pearl’s (2009, p. 202) *structural model semantics*.^22^ A comprehensive treatment of local regularities is not possible here (see Cavalcanti 2018; Pfeiffer et al. 2008), but the essentials of categorializing Pearl’s approach can be covered briefly.

We can begin with the definition of a *structural causal model*, as a triple $M = \langle B, E, F \rangle$ where,^23^

1. $B = \{b_{i}\ | \ i \in I \}$ is a set of “background”, “predetermined” or “exogenous” variables, intuitively conceived as things constant and *outside* of the model.
2. $E = \{e_{i}\ | \ i \in I \}$ is a set of “endogenous” variables that are determined by things *inside* the model, i.e., by $B \cup E$.
3. $F = \{ f_{i} \ | \ f_{i}{:}\, B \cup E \rightarrow e_{i}$ is a partial function$\}$ such that $F{:}\, B \rightarrow E$. Overall *F* is a mapping from background to endogenous variables.

This allows us to define a category $\mathfrak {Cau}$ having structural causal models as objects and morphisms defined components-wise on these as triples. That is, functions $g:B_{1} \rightarrow B_{2}$ and $h{:}\, E_{1} \rightarrow E_{2}$ define morphisms $f{:}\, M_{1} \rightarrow M_{2}$ of structural causal models provided the following diagram commutes. Pearl suggests thinking of individual $f_{i} \in F$ as specific mechanisms, and in like manner we might think of morphisms of structural causal models as *mechanism-order preserving maps*. Moreover, since each such model gives rise to a directed graph *G*(*M*), there is an evident mapping of this category into $\mathbf{Dgr}$ where morphisms are defined as usual. 
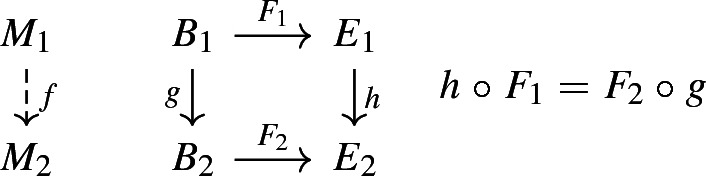


A *local structural causal model* is then a functor,

$$\begin{aligned} \mathcal {F}{:}\, \mathcal {T}_\mathbf{X }^{\,\,op} \rightarrow \mathfrak {Cau} \end{aligned}$$

To the end of modelling local causation, it sometimes makes sense to restrict our attention to the morphisms corresponding to “local actions” or “submodels” (see Pearl 2000, § 7.1.2). Essentially, these are just morphisms that are identities on *B* and *E*, but which allow certain $f_{i} \in F$ to become constant, effectively backgrounding them or setting them to a value specifying a local condition or an hypothetical change, including, in Pearl’s terminology, those “implied by counterfactual antecedents” (p. 204). Were the aim to model local regularities, under the assumption that these arise from local actions or local changes in mechanism, then the appropriate models are pre-sheaves $\mathcal {F}{:}\, \mathcal {T}_\mathbf{X }^{\,\,op} \rightarrow \mathfrak {Cau}_{M}$ having sections in the category $\mathfrak {Cau}_{M}$ of sub-models of some given “global” structural causal model *M*.

## Conclusion: why we should use sheaves

Thus far the aim has been to build up to the machinery necessary to model causation locally—the minimum required to give meaning to claims that *causation here* is different from *causation there*. Care has been taken *not* to place any constraints on the account other than those demanded of the background mathematics—the conditions on being a category, functor, mapping between pointed related sets or map of digraphs. In conclusion, to save something of the intuition that causation involves more than merely local relationships, that causation involves “global” relationships, I suggest that the additional constraints we *should* put on local causation are those required to specify pre-sheaf models as fully-fledged sheaves.^24^ Put another way, if we can provide some local model of counterfactuals or regularities as a presheaf $\mathcal {F}$ and we are aiming for a more global model of causation, then $\mathcal {F}$ should be a sheaf.^25^

Universal accounts of causation suffer persistent problems (Sects. 1 and 2), but to reject the universality of causation entirely seems to imply a world without causes or laws, since both seem to depend integrally on universal notions (Carroll 2016; Anjum and Mumford 2018). Indeed, a world where all causal goings-on are irregular, accidental, or monads, is philosophically dissatisfying. Faced with this dilemma, as Lewis (1973) says regarding the problems facing regularity analyses, “it is time to give up and try something else”.

My response is that the dilemma is a false one. We are not in the theoretical position of being forced to *entirely* accept or reject what is held dear about universal accounts of causation, since understanding causation as sheaf-like provides a middle way. Our dilemma can be reframed as a problem of keeping track of local causal variation and amalgamating compatible information when possible; our metaphysics then need only hold that there is a consistent way to do so. Between this Scylla and Charybdis, the view of causation as *sheaf-like* (and that therefore our models of causation should be *sheaves*) allows one to navigate more freely between these extremes.

Between the horns of causal universality (UA) and mere locality (LA), sheaves offer principles of intermediate strength, since they build in assumptions about how compatible information can be glued or concatenated together (Sect. 3.1). The *locality axiom* is vital to ensuring that sections of sheaves are uniquely determined locally and that the concatenation of compatible sections is unique. The *gluing axiom* of sheaves offers something *sui generis* to handle the problems facing universal accounts of causation.^26^ This is really a consequence of what sheaves offer to local truth, but extended up to causal truths. When granted ways of restricting our claims, further localizing them, and comparing these restrictions with intersecting locations, gluing allows us to come to more global claims by piecing together our local sections. Viewing causation as sheaf-like allows us to accept that under the best conditions (agreement of compatible sections) we can further globalize or universalize our causal claims, without assuming any claims are universal to begin with. That is, we can use gluability instead of universalizability as a criterion of genuine causal relationships.

A sheaf-like account of causation brings our ontology more in line with our science: both being epistemology *en plein air*. Doing ontology in the field, we are better off gluing compatible causal information together than assuming universality; better off sketching the local landscape than supposing what is indemonstrable in principle. Returning to the visual analogy (at the beginning of Sect. 3), by looking at specific sections of the sheaves of wheat in a field we may be unable to determine the overall direction of the wind. Indeed, there may be no consistent direction spanning the entire landscape. Nonetheless, we should be able to come to a more circumspect view of the weather, causation writ large, by examining sections covering greater and greater portions of the topography.

## Footnotes

1 A moderate position (suggested to me by Jacob Stegenga), is that all extant accounts of causation can be reasonably presented in a way that is committed to UA.

2 Of course, there are other accounts of causation, such as Woodward’s (2005) interventionist account, and correspondingly other models of causation (see Fig. 2, Sect. 3.2). I confine my discussion to this broad split between counterfactual and regularity accounts for tractability. On the other hand, the counterfactual and regularity accounts are not wholly distinct—both Lewis and Pearl occasionally deploy both regularity and counterfactual notions (e.g. Pearl 2009 p. 389). Moreover, Woodward’s (2005) account of necessary and sufficient conditions for causal relationships is both evidenced by regularities and defined in counterfactual terms (p. 250), and Pearl’s (1995, p. 670) account of causal diagrams is explicitly stated in terms of interventions. I separate them here (in Sects. 3.2.1, 3.2.2) since it is easier to discuss their models separately.

3 Of course, this is not the only interpretation, but it is a plausible one. Hume might indeed be re-read today by replacing ’where’ with ‘where and when’, or he may simply have had in mind something similar to ‘just in case’ or ‘such that’. The point is that reading ’where’ as specifying a location does not require any re-reading at all.

4 Which are Kripke-models, provided a relation capturing comparative similarity (Garson 2000), discussed in (Sect. 3.2.1).

5 There are of course other sorts of underdetermination, such as those arising between competing theories with distinct non-empirical commitments, that are not addressed by moving to a local account of causation.

6 Moreover, the making of observations requires instrumentation, the causal structure of which must be *assumed* not to vary between locations since, as Whitehead (2010) notes, there can be no “infinite regress of instrumentation”.

7 Of course, if we are in a position to empirically distinguish **V** and **U** then there must at least be some observable difference *de se*, such as that they are observable from different perspectives or by different observers, but—barring certain causation-observer interdependencies—these will not prevent us from having precisely identical observations about **V** and **U** *de re*.

8 They do not, however, require that we sacrifice our ability to make inferences about unobserved locations, provided those locations bear certain topological relationships with ones in which we have previously established causal relationships. This is discussed in Sect. 4, with background provided in Sect. 3.1.

9 That is **B**, the causal nexus, background causal conditions, or “causal machine” in Cartwright’s (2004) sense.

10 Other explanations could be *de se*, relying on the fallibility of the investigator.

11 Woodward’s (2005) manipulability or interventionist theory of causation—which has both regularity and counterfactual aspects—is perhaps particularly well-suited to modifications required by a local account of causation. For example, on that account, “generalization can be invariant within a domain even though it has exceptions outside that domain or holds only within a limited spatiotemporal region” (p. 240).

12 See Seebach et al. (1970) for history and examples.

13 Only the essentials of sheaf theory are required. These are explained in sufficient detail in Sect. 3.1.

14 See Abramsky and Sadrzadeh (2014) for a sheaf-theoretic approach to semantics in natural language.

15 Though this should not be taken as a preference for or endorsement of any of the axioms of the basic system appearing in Simons (2006).

16 Let us say that providing a truth-value distribution or its transpose is “doing local logic”, and that evaluating truth-value functions at $\tau$ is just “doing logic locally”. An analogous distinction, between “doing local science” and “doing science locally”, appears later (Sect. 3.2).

17 Though, the analysis of systems that can be formed with these resources is not the aim of the present connection

18 The interested reader may compare Goldblatt (1984), Tennison (1975), Lovering (2010), and *Introduction to Schemes*, by G. Ellingsrud and J. C. Ottem

19 Provided we could augment our account of conditional probabilities between events with nominals, or event names that are true always and only in given nations or months (e.g. ‘England’, ‘Norway’, and ‘January’), this point can be succinctly rephrased. The conditional probability, $Pr(\text {Frost} \ | \ \text {January} \ \& \ \text {England})$ does not in general determine $Pr(\text {Frost} \ | \ \text {January} \ \& \ \text {Norway})$.

20 The answer to this question is of course underdetermined and may at times require only such non-iconoclastic maxims as “be circumspect” or “be attentive to local details.” The problem with this deflation is that such maxims themselves require some notion of locality to be part of rigorous science, e.g. we should like to know how far one must look around one’s location of experiment before one has been satisfactorily circumspect.

21 Feminist philosophers of science, attentive to the local social conditions of scientific knowledge production generally, are perhaps the vanguard of a philosophy of local science as a *local epistemology* (Longino and Lennon 1997).

22 As well as complementing the local counterfactual analysis of causation, the causal graphs approach helps achieve one of the desiderata—highlighted especially in spatial epidemiology (Jacquez 2000)—of local models: a move from models of (probabilistic) data to models of process.

23 Ideally, these models would be inferred from empirically derived conditional probability distributions. Also note: the notation in Pearl (2009) has been modified for consistency here.

24 This is not to say that we should always require our causal models to be sheaves—presheaves that are not sheaves can still serve a valuable role when full sheaf structure is unavailable, as may obtain in the case of certain quantum phenomena (Abramsky and Brandenburger 2011).

25 Similarly, if $\mathcal {F}$ is not a sheaf, the aim of obtaining a global model of causation implies that we *should* model causation by its sheafification $\mathcal {F}^{\sharp }$.

26 Separated presheaves may have a place in causal modelling, but they are insufficient to deal with problems of underdetermination.
